# Editorial: Soilless Culture for Vegetative Biomass Production and Specialized Metabolites: Medicinal, Aromatic, and Edible Plants

**DOI:** 10.3389/fpls.2022.887487

**Published:** 2022-04-07

**Authors:** Rita Maggini, Nikolaos Tzortzakis, Christopher Jon Currey

**Affiliations:** ^1^Department of Agriculture, Food and Environment, University of Pisa, Pisa, Italy; ^2^Department of Agricultural Sciences, Biotechnology and Food Science, Cyprus University of Technology, Limassol, Cyprus; ^3^Department of Horticulture, Iowa State University, Ames, IA, United States

**Keywords:** bioactive compounds, controlled environment, hydroponics, nutrient solution, wild species

## Introduction

Due to their content of bioactive compounds, medicinal, aromatic and edible plants can be regarded either as functional foods or as plant material for the extraction of pharmaceutical molecules (Garcia-Oliveira et al., 2021). Since many years, the increasing market demand for healthy foods or plant-derived health products, such as natural remedies and food supplements, has directed food and pharmaceutical industries toward the exploitation of the bioactive properties of these species (Lubbe and Verpoorte, 2011). Typically, bioactive compounds are secondary metabolites involved in the mechanisms of adaptation of plants to their natural environment. Therefore, the synthesis and accumulation of these specialized molecules in plant tissues are influenced by the nutritional and environmental conditions experienced by plants during their growth. The cultivation of medicinal and aromatic plants, as well as the commercial production of edible species, is an effective means to sustain the market demand for natural healthy products and enlarge the market offer of specialty crops (Canter et al., 2005; Maggini et al., 2021).

Soilless greenhouse culture is particularly well-suited for the cultivation of these species (Dorais et al., 2001), as it allows to avoid plant contamination problems linked to the agricultural soil and ensures plant mineral nutrition though a nutrient solution that contains all the necessary macro- and micro-elements. Soilless culture encompasses greenhouse hydroponics and substrate culture, along with new non-conventional integrated growing systems such as aquaponics, which combines hydroponic cultivation with fish farming. With both hydroponics and substrate culture, the plants are subjected to nutritional and environmental conditions that can be appropriately tailored to match the needs for plant growing and optimize their content of secondary metabolites (Chrysargyris and Tzortzakis, 2021). Research on this topic will contribute to elucidate the physiological basis of the adaptability of medicinal, aromatic and edible plants to soilless cultivation.

## Soilless Culture for Vegetative Biomass Production and Specialized Metabolites

The use of new agricultural technologies such as soilless cultivation systems is a valuable approach to medicinal plant production. Sweet basil (*Ocimum basilicum* L.) is one of the most produced aromatic herbs in the world, exploiting hydroponic systems. Kolega et al. justified the beneficial effects of nutrient solution management and the application of plant-growth-promoting rhizobacteria, enhancing the nutritional value such as the content of arginine, of “Red Rubin” basil variety. Moreover, Ciriello, Formisano, El-Nakhel, et al. discussed the role of nutrients such as calcium application and effectiveness toward the alleviation of salinity-induced stress in basil, highlighting the impacts of the ratio of different salt types that might induce differential responses in basil plant metabolism. Indeed, nutrient solution management in hydroponics is not the sole factor for high quality production of crops, while altering the radiation intensity in controlled environments can influence the biosynthetic pathways of volatile organic compounds, including those of terpenoids and phenylpropanoids. In that sense, Walters et al. studied the effects of radiation intensity and CO_2_ concentration on sweet basil, and correlated consumers preference characteristics with levels of 1,8-cineole, eugenol, and linalool as observed in basil grown under a radiation intensity of 200 mmol m^−2^ s^−1^. Additionally, Ciriello, Formisano, Soteriou, et al. discussed the effects of plant density, season and basil cultivars on yield and secondary metabolites modulation, with significant increases in yield, phenolic acids, and favored eucalyptol and 1-octen-3-ol accumulation.

Corrado et al. studied the effects of the isosmotic variations of the concentration of three macrocations (K, Ca, and Mg) in lettuce (*Lactuca sativa* L.), which were reflected in changes in plant morphology, physiology, quality and functional properties of the edible product. Moreover, Zhang et al. reported an increased alkaloidal content in *Mitragyna speciosa* (kratom) as affected by the nutrient supply, as low to medium rates of fertilizer maximized the concentration of speciogynine, corynantheidine, and isocorynantheidine per leaf dry mass, suggesting a promotion of nitrogen allocation for secondary metabolism and thus their synthesis under such condition.

The drug-type *Cannabis sativa* L., a crop with increasing global importance, has been evaluated by Yep et al. regarding the effects of salinity (NaCl) from 1 to 40 mM, when plants were grown in hydroponics and aquaponic solutions during flowering stage. It was found that cannabinoids decreased linearly with increasing NaCl concentration, while 40 mM NaCl-salinity negative impacts on plant growth and physiological response were evidenced in hydroponic system but not in aquaponics, suggesting putative exploitations for the latter.

Elicitation mediated aeroponic cultivation for targeted metabolites was examined by Partap et al. for the production of *Valeriana jatamansi* using elicitors, such as yeast extract and methyl jasmonate. The results revealed that, besides roots, leaves could also be utilized as a prominent alternative source for valerenic acid and its derivatives, and elicitors application in medicinal plants under aeroponic cultivation might be exploited for its pharmaceutical applications and commercial-scale production in less duration to meet the unmet demand of the industries.

## Conclusions

The high-standard scientific studies collected in this Research Topic, demonstrate that the synthesis and accumulation of bioactive compounds in plant tissues can be accomplished with soilless culture through the modulation of mineral nutrition and control of the growing conditions. Both the composition of the nutrient solution and the environmental parameters affect tissue quality and biomass production. Therefore, correct management of these parameters is crucial for the development of proper growing practices. Different specific techniques have been examined in this Topic, including a change in mineral nutrition, the application of light control, or the use of microorganisms to modify the root microenvironment. All these systems provide an insight into the physiological and nutritional conditions of plants and can help to optimize biomass yield and tissue quality. We believe that the contributions to this Research Topic show innovative and sustainable approaches to the soilless cultivation of medicinal and aromatic plants that can address both environmental and health concerns. We expect that this collection of high-level scientific papers will promote further discussion about this topic and open a perspective for the application of soilless growing methods to other medicinal, aromatic or edible species with ethnobotanical interest. These species may exhibit a wide range of bioactive properties that are still partly uncharacterized or unexploited.

## Author Contributions

RM wrote the first draft (sections Introduction and Conclusions). NT wrote the first draft (section Soilless Culture for Vegetative Biomass Production and Specialized Metabolites). RM, NT, and CC revised the manuscript. All authors contributed to the article and approved the submitted version.

## Conflict of Interest

The authors declare that the research was conducted in the absence of any commercial or financial relationships that could be construed as a potential conflict of interest.

## Publisher's Note

All claims expressed in this article are solely those of the authors and do not necessarily represent those of their affiliated organizations, or those of the publisher, the editors and the reviewers. Any product that may be evaluated in this article, or claim that may be made by its manufacturer, is not guaranteed or endorsed by the publisher.
